# Diversity attitudes and sensitivity of employees and leaders in the German STEM-sector

**DOI:** 10.3389/fpsyg.2022.960163

**Published:** 2022-08-22

**Authors:** Petia Genkova, Henrik Schreiber

**Affiliations:** Department of Economic and Social Sciences, University of Applied Sciences Osnabrück, Osnabrück, Germany

**Keywords:** diversity, STEM sector, discrimination, culture, migration, age, older migrants

## Abstract

The increasing diversity of cultural backgrounds, migration status and age is shaping modern working life. Previous research demonstrated that the attitudes and competences of team members and leaders toward diversity play a crucial role in utilizing the potential of heterogeneous teams and preventing detrimental behavior and discrimination. But even though leaders appear to be key figures in the process of diversity management, their perception of diversity as well as related challenges and chances are poorly investigated. The current paper aims to contribute to the understanding of leaders’ perspective on and role in diversity management building on a comparative analysis of two explorative qualitative studies with 16 employees and 22 leaders. The overall research questions are how employees and leaders perceive diversity of culture and age from their specific point of view, which experiences are likely to contribute to their opinion on and perception of diversity and in how far do employees and leaders differ in these aspects. Participants come from the German sector of science, technology, engineering and mathematics (STEM), which is the most internationalized and least psychologically investigated sector in Germany. The results show that employees are aware of the topic of diversity in general but have poor competences in dealing with diversity in their daily work life. This seems to be associated with a lack of experience with intercultural interaction and a lack of support from the respective organizations/leaders. We further found that individuals with a migration background do not show any signs of stereotype threat rather than expressing a feeling of being isolated from employees without a migration background. By comparing perspectives of leaders and employees, the current study contributes to the understanding of the processes underlying the experiences of inequalities of migrants and experiences of intercultural miscommunication and faultlines of people without migration background. Theoretical and practical implications are discussed.

## Introduction

Structures that are characterized by a high level of heterogeneity among their members are successful if they succeed in utilizing the given diversity of skills and perspectives (van Dick and Stegmann, 2016). This is the aim of the approaches and methods summarized under the term diversity management. On the one hand, depending on how diversity is dealt with in work groups, age and cultural diversity lead to better group performance (Stegmann, 2011; Wang et al., 2019). On the other hand, intergroup prejudice might deprave successful coworking of individuals with different cultural backgrounds and from different age cohorts.

The utilization of diversity in the workplace is naturally linked to the interaction of the individuals involved. Previous studies, in which coworking in diverse groups was understood as “intergroup-behavior,” demonstrate the central role of employees’ attitudes toward and perception of diversity for fairness and performance outcomes in diverse teams (van Knippenberg et al., 2004; van Dick and Stegmann, 2016). Furthermore, managers play a crucial role in the implementation of diversity management. While has the task of developing a general strategy and public position, senior and middle managers are required to exemplify this strategy by demonstrating openness and flexibility, sanctioning inappropriate behavior and selecting employees based on fair and valid criteria (Rosken, 2016). They are therefore also described as multipliers (Wildermuth and Gray, 2005). Previous studies on leadership and diversity indicate that many German managers do not recognize the importance of diversity (Bader et al., 2019; Genkova and Schreiber, 2019), compared to other countries (Buttner et al., 2006; Eger and Indruchová, 2014; Madera et al., 2017). Quantitative studies demonstrate that manager’s attitudes towards diversity are predicted by organizational climate and organizations affirmative action (Buttner et al., 2006; Biswas et al., 2021). While these studies underline the importance of such actions comprehensively, they fail to investigate managers specific perspective on and perception of diversity or if they differ from employees’ perspectives. Accordingly, explorative research is needed for groups of managers (e.g., in a special sector) in the first place in order to investigate and support managers dealing with diversity.

Studies by Krell (2013) show that in Germany most diversity-related challenges regard the dimensions gender, age, and cultural background (Genkova and Ringeisen, 2016). One sector, to which diversity-management is thus especially important is the German STEM-sector (sciences, technology, engineering, mathematics). It is one of the most important sectors in German economy and the most internationalized sector as well. According to the Institute of German Economics, the STEM sector is characterized by high international mobility on the labor market, and a high proportion (19%) of foreign experts (Anger et al., 2018). At the same time, Germany is experiencing demographic change, related to a higher proportion of older workforce. While gender-diversity in the STEM sector received extensive attention in public discourse and research lately (Anger et al., 2018), perspectives on and consequences of age and cultural diversity remain mostly unattended. Recent reports indicate that handling of diversity is neither established in curricula (Koller et al., 2017; Auferkorte-Michaelis and Linde, 2018) nor in public awareness (Anger et al., 2018). Thus, investigating culture and age diversity-management in the German STEM-sector appears especially promising.

We therefore explore the questions how STEM employees and leaders in Germany perceive diversity of culture and age from their specific points of view, which experiences are likely to contribute to their opinion on and perception of diversity, and in how far do employees and leaders differ in these aspects. By revealing social attitudes, challenges, and chances of STEM leaders (which are important stakeholders in decreasing discrimination at the workplace), we strive to contribute to the examination of discrimination and potentials of migrants aging in place. The following section thus describes phenomena related to diversity of cultural background and age, followed by explanations on the management of diversity and the role of leadership.

### Diversity and diversity management

The central topic of diversity management is the variety of members in an organization. In this sense, diversity refers to personal variety, i.e., the similarities and differences between individuals (Krell, 2013). Diversity includes both obvious and barely perceptible stable traits, such as religion, sexual orientation, cultural values, as well as changing characteristics such as language and competence (van Dick and Stegmann, 2016). While all those characteristics are more or less relevant, some dimensions of diversity are especially meaningful due to their importance for an individual’s identity as well as the challenges and chances related to it.

Research on challenges and chances of cultural and age diversity mostly based on two underlying theoretical perspectives: the information-processing perspective and the social-identity approach (Schneid et al., 2016). The information-processing perspective suggests that diversity is linked to application and better elaboration of relevant information and thus to greater performances of diverse teams. The core of the social-identity approach is that the formation of subgroups based on the common expression of relevant attributes (e. g. in age or cultural background) leads to conflicts and hostile behavior (van Dick and Stegmann, 2016).

Culture and age are both relevant aspects of social-identity for an individual and are used to categorize themselves and others, with a greater emphasis on subjectively relevant differences between groups (social-identity approach; Tajfel and Turner, 1979). Thereby, culture is defined as a psychological orientation system that provides identity through norms for perception, thinking and acting (Thomas and Simon, 2007). The moment the belonging to a certain cultural or age-group becomes more salient, or subjectively more relevant, people tend to see themselves and others less as individuals and more as prototypical representatives of a single group (van Dick and Stegmann, 2016).

The categorization elaboration model (CEM, van Knippenberg et al., 2004) is based on the understanding of identity in line with the social-identity approach and was the first model that could explain the performance differences between different diverse teams in a replicable way (van Dick and Stegmann, 2016). The core assumption of the CEM is that groups with diverse members have a higher potential for perspectives, knowledge or general information if they work together efficiently with the corresponding subgroups. Ideally, the diversity of information should lead to more in-depth information elaboration and thus to better thought-out or more innovative solutions. Subgroup formation (subjectively relevant group characteristics and salience of this group) then leads to negative affective consequences and consequently to poorer performance if the subgroups have a hostile relationship with one another. According to this model, this occurs when the subgroups do not recognize each other or their respective contribution, i.e., threaten their identity (van Dick and Stegmann, 2016).

Beyond behaviors directly related to organizational performance, research reveals that subgroup formation based on age and cultural background is related to severe consequences for fair chances and appropriate intergroup behavior (Kunze et al., 2013). Individuals with cultural backgrounds other than the company majority as well as very young or old employees might be disadvantaged due to discrimination and hostility from colleagues and managers (Sanchez, 2018), but also by limited access to career options, social capital and networks (Ferris et al., 1993; Kunze et al., 2013). Especially when there are strong expectations toward migration status or age for holding a certain position, individuals might experience disadvantages during selection/promotion or discrimination in the workplace (Ferris et al., 1993; Kunze et al., 2013). Experiences of discrimination overtake victims’ cognitive resources and disturb individual performance (Walker et al., 2021). Consequences of experiences of discrimination may also include minority stress (Goldman et al., 2008), depression, anxiety, or somatization (Torres et al., 2012) as well as less commitment to the organization (Cox, 1991). Thereby, intersectional perspectives emphasize that, in average, older people with migration background are more disadvantaged than younger people with migration background in the life-course compared to non-immigrants of the same age, respectively. This might indicate that older employees with migration background are excluded more strongly from organizational networks and have been disadvantaged in selection and promotion processes in the past (Stypińska and Gordo, 2018). While anglophone literature often focuses on race diversity when considering intergroup relations and migration (Yadav and Lenka, 2020), previous studies in Germany revealed that race diversity plays a smaller role in Germany. While there is race diversity and racism in Germany, many relevant cultural minority groups do not differ in appearance (e. g. former soviet-union immigrants), but experience discrimination and challenge of intercultural interaction as well (Krell, 2013; Genkova and Ringeisen, 2016).

### Diversity management and leadership

van Dick and Stegmann (2016) conclude that the main challenge for modern diverse teams is not the diversity of their members, nor the inevitable subjective relevance of differences—fault lines (break lines)—between subgroups. Rather, the most important task of diversity management is to replace any threatening relationship between the groups with a productive and meaningful relationship. This requires a positive, shared attitude toward diversity, awareness for heterogeneity and challenges through diversity as well as skills for cooperating. A superordinate identity that increases cohesion and commitment is fundamental to collaboration (van Knippenberg and Schie, 2000). Based on the in-group projection hypothesis (Mummendey and Wenzel, 1999), this also requires an awareness that the superordinate group, the team or the organization is not culturally homogeneous, but becomes what it is through the multitude of different perspectives. Moreover, Gutentag et al. (2018) show that the basis for diversity-sensitive behavior is a differentiated awareness of cultural diversity. This runs on a spectrum between the culture-blind (assuming there are no cultural differences, all people are equal) and colorful (accounting for cultural differences; Cox, 1991) perspectives, which are used both by organizations and people. In addition, individual attitudes toward the instrumentality of diversity are crucial (Stegmann, 2011). The term diversity beliefs was originally introduced by van Knippenberg et al. (2003) to denote individual beliefs that diversity is beneficial to a group.

Those attitudes toward diversity among employees are shaped by the behaviors of leaders (for an overview see Genkova, 2019). Ashikali and Groeneveld (2015) use a large study with more than 10,000 participants to show that the perception of fair selection processes by the employees, the perception of appreciation and a conscious, competent handling of diversity by managers are significantly positively associated with the diversity attitudes and the commitment of the respective employees. This relationship is partially mediated by a transformational leadership style (Ashikali and Groeneveld, 2015). They explain this relationship by the fact that employees in general, and in particular under transformational leadership, adopt attitudes and behaviors in the sense of developing a diversity culture. The diversity culture, i.e., a socially shared set of opinions and behaviors (Schein, 2010) with regard to the assessment of how to deal with diversity, is a central predictor of the performance of heterogeneous work groups (Kundu and Mor, 2017) and managers evaluation of diversity (Bader et al., 2019; Biswas et al., 2021). Supportive organizational structures and a diversity-promoting climate are thus particularly beneficial, which can reduce intolerance and promote openness toward “otherness” (Biswas et al., 2021).

Previous meta-analyses by Stegmann (2011) and Wang et al. (2019) show that cultural diversity can have a positive effect on team performance depending on participant attitudes toward diversity. While Schneid et al. (2016) could not find a consistent effect of age diversity on team performance, Kunze et al. (2013) show that managers’ diversity attitudes and diversity-friendly HR-policies predict diverse team performance *via* age-related diversity culture.

Managers are generally caught between understanding the connections and relevance of diversity management, considering their other management tasks and possible professional tasks, developing sensitivity and possibly leaving their own animosity and insecurities behind (Ashikali and Groeneveld, 2015; Genkova, 2019; McCallaghan et al., 2020). Research on the implementation and success of diversity management emphasizes that in order to actually reduce discriminating structures and behaviors, a common understanding is needed of which behaviors and perspectives are considered discriminating (Auferkorte-Michaelis and Linde, 2018). Also, people with migration background as well as younger and older employees might hold specific needs for support in order to overcome established forms of discrimination. However, you cannot change what you do not see, and this kind of sensitivity toward diversity is not widespread among German companies (Charta der Vielfalt, 2019; Genkova and Schreiber, 2019). Also, corporate strategies might differ strongly from employees’ actual needs. While managers orientation toward diversity is correlated with organization diversity actions (Buttner et al., 2006), this might cause a misfit between managers’ and employees’ perceptions of diversity, decreasing efficiency of diversity actions and maintaining barriers. Thus, managers are required to be not only aware or sensitive toward general forms of diversity, but they need to develop a good knowledge of challenges and strengths for their company and their team.

In summary, efficient diversity-management requires in-depth understanding of managers’ and employees’ perception of and attitudes toward diversity. Previous studies on this topic focused mostly on examining predictors of managers’ awareness of general issues of diversity (Buttner et al., 2006; Eger and Indruchová, 2014; Genkova and Schreiber, 2019) and evaluation of diversity (positive/negative; Bissles et al., 2001; Bader et al., 2019), rather than investigating the specific meaning of and perspectives on diversity that might root in an in-depth or superficial awareness of diversity. As described in the introduction, these insights are particularly crucial for the STEM industry (Anger et al., 2018). Although it can be assumed that the skills, attitudes and perspectives of STEM graduates differ from those of people with a degree in the humanities or economics (Canagarajah, 2018), there are no differentiated results for managers in the STEM industry in front. The aim of this study is therefore to contribute to a better understanding of the attitudes toward and perception of diversity of managers in STEM professions. To allow identification of discrepancies in the perception of needs, conflicts and power asymmetries, we analyze employees’ and managers’ views on diversity of age and cultural background comparatively. The following key questions were formulated accordingly:

1. What are the attitudes toward diversity among the STEM employees and leaders surveyed?
2. Which experiences and perspectives are particularly relevant for their attitudes toward diversity?
3. In how far do attitudes of surveyed managers and employees differ?

## Methodology

In order to explore those research questions, we used semi-structured qualitative interviews in order to provide a picture of the subjective theories (Hilmer, 1969) of managers and employees from the STEM sector on diversity. The interview guide contained a total of 58 questions. To ensure conceptual equivalence and comparability (Genkova, 2019) across the subgroups (managers and employees with and without a migration background, with a lot and little experience), the completed interview key questions were discussed by several experts on diversity with and without a migration background and released after minor changes with regard to general formulations.

The interviews took place between July 2019 and March 2020. Managers and employees from various companies who have a degree in MINT subjects and work in this field were acquired for telephone interviews *via* scientific and economic networks. The acquisition aimed to reach participants from different age groups, with and without migration background, different sexes, with different hierarchical status and from different types of companies. In accordance with the privacy policy, explicit consent was obtained for the interviews to be recorded and used for academic purposes only. The interviews lasted between 20 and 45 min. No incentive was paid. The recorded interviews were transcribed and analyzed according to the inductive qualitative content analysis described by Mayring (2019). Thereby, categories were formed inductively by paraphrasing (removing filler-phrases, slang, etc.), generalizing (increasing abstraction level) and then categorizing participant statements (summarizing generalized statements) in order to allow for a high level of abstraction while sticking closely to the original statements. To verify the identified categories, three diversity experts from universities discussed the findings. This corresponds to a triangulation procedure proposed by Bengtsson (2016).

### Participants

A total of 22 managers and 16 employees from various German companies were interviewed. The aim was to obtain a heterogeneous sample regarding age, sex, migration status, position, and size of the affiliated company. Managers were between 26 and 69 years old (M = 49), while employees were between 24 and 46 years old (M = 34). Seven respondents were female, and 31 were male. All respondents had a degree in a STEM subject. Eight respondents had a migration background (according to the definition of Kemper (2010): people who immigrated to Germany themselves or at least one of their parents). Five respondents (23%) said they worked in a small company (up to 50 employees), 16 (36%) in a medium-sized company (up to 250 employees) and 17 (41%) in a large one companies with more than 250 employees. All participants were working in some form of team-structure requiring interpersonal interaction and coordination on a daily base (e.g., project based work or agile work). From the 22 managers, ten classified themselves in the middle management level, 12 in the upper management level.

## Results

The following section presents the results of the current study, reporting frequencies of answer categories and pattern, relationships between answers, and illustrating conclusions with quotes. Thereby, we roughly follow the order of the research questions. However, experiences and relevant perspectives might be better understood by examining contrasts between employees and managers. We thus start with an analysis of the given attitudes and shift toward a comparison more and more when looking at the related experiences and social environment factors.

In order to answer the question, which attitudes toward cultural and age diversity exist among the participants, various questions were asked, which intended to reveal the perceived and assumed advantages and disadvantages of cultural and age diversity in companies. While all interviewees were able to express an opinion about diversity, the perspectives, underlying assumptions and experiences with aspects of diversity differed between participants. Around two-thirds of the interviewees referred to cultural diversity in their answers, giving examples like team members with migration background or international cooperation with partners and customers. Not surprisingly, nearly all of the participants with migration background gave answers in the category of intercultural diversity, but they rarely talked about other issues of diversity. One-third of the participants referred to fields of specialization as a diversity characteristic; for example: “*To develop an app, one needs a diverse team, including different competences in programming and probably in user-interface design*.” There were also participants with migration background who gave answers in this category. References to age groups, gender, diverse opinions and worldviews were made three times each. Considering the number of mentions as an indicator for the relevance of the respective dimension, fewer participants were aware of the issues age, gender and opinions, at least compared to the issue of cultural diversity. Besides focusing on certain dimensions of diversity, answers varied in their degree of elaboration.

Five managers and three employees gave several answers in which they discussed various aspects of diversity, advantages, and disadvantages in an elaborated way (categories: work motivation, openness, working more efficiently, promoting integration, mutual learning, additional skills). They also expressed a generally open perspective toward hierarchy and roles, referring to age, culture, and other dimensions. For example, one participant emphasized the role of diversity in the corporate climate and employee performance. “(…) *there are different people every day who have different backgrounds, sometimes cultural backgrounds, but simply also come from different life situations. And if we are open, ‘um’/ well, if we already learn that internally, (..), then we can also use these positive experiences to be open to external parties, i.e., to our customers, and learn from them.*” Those participants also expressed the belief that the challenges and stress levels of employees with a migration background differ and describe both opportunities and risks for people with a migration background. Five of them had a migration background themselves. None of the participants expressed the belief that challenges for older and younger employees differ. However, most younger employees and managers described that they sometimes have to prove themselves in order to be accepted in their professional role. The younger and older participants who demonstrated a higher sensitivity to diversity also stated that they experience the cooperation of older and younger employees sometimes as challenging. Older workers, in particular, reported that they find it challenging to rely on new ways of working while at the same time contributing their expertise.

In contrast, 12 of the remaining respondents could not give any or only a brief answer referring to direct consequences of diversity (categories: access to other markets (international and to migrants), languages, other perspectives, working methods). Uncertainty seemed to show itself in short, choppy responses that took up more general positions, sometimes combined with requests to continue the interview (e.g., “Um, diversity brings advantages (..) Especially with project work. Next question, please!”). Later in the interviews, those participants focused more on preventing individual difficulties that arise from intercultural misunderstandings. Many of them also stated that learning German was the most important thing for migrants to do in their company. However, when asked when and where a migrant has the opportunity to learn German in their company, they emphasized that this should be accomplished during free-time. Accordingly, more than half of the interviewees without a migration background expressed the opinion that it is the best to ignore one’s own cultural peculiarities, to hide them or to show them only cautiously in appropriate situations to avoid problems. A similar assimilative perspective was expressed when referring to age diversity. Younger, less sensitive participants reported that it is challenging for them to prevail against established concepts. Older, less open-minded or sensitive participants, however, expressed no awareness of such conflicts. It was rather considered as a natural asymmetry, that younger employees want to establish new concepts, but due to a natural lack of experience their attitudes and perspectives usually do not work out.

In summary some participants displayed high levels of awareness for diversity irrespective of migration status or age. However, the majority of participants were rather insensitive toward diversity and related challenges and chances. This came along with a strong focus on assimilation, demanding for homogeneity and commitment to established working and role models. Additional challenges for younger or older people and employees with immigration background were not considered either. Moreover, most participants were not aware of the opportunity to actively influence diversity or diversity culture.

Regardless of being sensitive toward diversity or not, employees and managers differed in how they defined their respective in-group from which they looked at diversity. While employees answered questions from an individual perspective exclusively (what does diversity do to me?), managers mostly referred to an organizational perspective. The conclusion seems obvious that one’s own negative attitudes, or one’s own insecurity and lack of skills are legitimized by higher authorities:

"*Yes, well, the management levels would certainly not accept it if employees align their carpet towards Mecca. In a German family company, German-run, that is not acceptable from the management, so: they would never do that*”.

In contrast, 17 of the managers surveyed expressed the opinion at various points in the interview that “*everyone is equal*” and—in six cases—that it is therefore particularly important to treat everyone equally, which is why no special actions are taken to support cultural diversity and equality. A closer look at the interviews shows that most of the interviewees act as representatives of their respective company and justify established social structures and norms. Those norms refer to thinking and acting, but also to age and migration background. All but two of the managers argue from the perspective of the company when it comes to personnel decisions (e.g., “*We have not done that much there yet; well, anyone can come to us*.”), or, for example, about dealing with errors (e.g., “*This is how we handle it…*”). The more frequently there are indications of a strong identification, the more the managers seem to see themselves as typical representatives of the company, especially with regard to the cultural background. Deviations, especially cultural deviations, seem to be viewed more critically when identification is high:

"Apart from professional suitability, what criteria do you use to select your academic staff?” “*EDP also means communication, and these are not just any tasks that have to be done in the back room, but you have to be able to talk and communicate with people. And we're a German family business, so yes, I'll say it, we're actually very limited locally. We do have branches in France and Poland, but German is definitely spoken in the management positions there too.*”

Those participants also expressed that people with a migration background and of the same age got along best with each other. This also applies to those managers who have a culturally heterogeneous workforce, or who often interact internationally. No such in-group projection can be observed for the four respondents who not only see abstract advantages in cultural diversity, but also see cultural diversity as a concrete strength of their own company.

Moreover, the actual experiences with diversity differ between employees and managers. While experiences of age diversity (fit to role models, ageism, prejudice, different perspectives and expertise) were very similar, experiences with cultural diversity differed strongly. First of all, there was a difference in the attitudes of participants who work a lot with international partners, clients or colleagues and those who do not. Those who operate internationally a lot, especially employees and managers from the IT sector and particularly large companies, often have a culture-blind attitude, but at the same time, have concrete strategies for dealing with cultural diversity. These respondents with many international contacts focused very much on the interaction with foreigners, while second-generation migrants are considered less relevant. Thereby, the focus was less on understanding intercultural differences, but rather on avoiding individual pitfalls. In three cases, managers emphasized an agile way of working and mentioned the Scrum framework as well, which provides for equal treatment of all team members. Agile working summarizes an extremely popular set of methodological frameworks that aim to enable efficient and flexible project management in the context of a volatile, uncertain, complex and ambiguos environment (VUCA, Schwaber and Sutherland, 2020). However, there seems to be no awareness within the company of issues relating to the proactive handling of cultural diversity.

Managers and employees who interact only slightly with international contacts or people with a migration background in their daily work (although the company can certainly serve international markets) show a more negative attitude toward cultural diversity. All managers who have the impression that cultural subgroups have formed in their companies have very little contact with people with a migration background in their everyday work. At the same time, these leaders were far less sure of what cross-cultural leadership requires and what immigrant and non-immigrant people can do to interact successfully. It is assumed that people with a migration background must learn German in any case.

On average, employees reported more interactions with employees, customers or clients with migration background. While less regular contact was also associated with negative attitudes, more experiences were not necessarily associated with better attitudes. Employees stated that they experienced very stressful or challenging situations during their first intercultural experiences or even later. It became apparent that they felt a lack of support during these experiences, as they felt ill-prepared and could not ask anyone with more expertise. Challenges thereby referred to misunderstandings or conflicts based on different understandings of hierarchy or working style. During interaction with employees with migration backgrounds, challenges also included language problems, misunderstandings and prejudices. Managers partially reported similar experiences; however, most of them (except three) showed no awareness that employees might need support to deal with cultural diversity. While most managers demonstrated that they consider cultural diversity as an obstacle that can be solved or ignored easily, employees were exposed much more intensely to the challenges of intercultural interaction and discrimination in the workplace. However, they report a lack of support, which might be explained by the managers’ lack of knowledge.

In contrast to this result, all managers except one were convinced that they were good at managing a diverse workforce and reacted sufficiently sensitively to the employees. Managers with a migration background did not differ in their response behavior from managers without a migration background. Although they were more sensitive than most other respondents, they did not necessarily seem to think in a more nuanced way about the issue. This could be an indication that while sensitivity is a necessary part of dealing competently with diversity, knowledge and “practical skills” may also be required. This coincides with the statements made by 11 of those surveyed that junior managers often have problems in this area. Five explicitly point out that they see this as a deficit in university education:

“*More tools, more education. I also said at the beginning that [diversity] leadership is practically non-existent at the university, at least I didn't have it as a subject at all, not even as an elective. And I get that mirrored by other people too. And when I think about the things I've learned in recent years, take Friedemann Schulz von Thun, his peer one model, or the different levels of communication and so on and so forth, all of these are actually basics, so really basics that you would normally have to pack in, let me say, the fifth to tenth grade. Because that's just incredibly important in further progress. And management simply has to go to universities, and that has to be one of the most important subjects, because afterwards you have to deal with people, everywhere.*”

This statement goes in line with the result that employees feel badly prepared and not sufficiently supported. Those who particularly emphasized this point in the course of the interviews have mostly completed various training courses in “soft skills” on their own, or with the support of their company. Managers who rarely attend these trainings have little awareness of the fact that employees and managers can be prepared for intercultural leadership. The fact that this could be the standard in other departments is not mentioned in any of the interviews, although it is repeatedly pointed out that too little is actually known about it.

## Discussion

The results show that only a small proportion of employees and managers of German STEM companies are aware of the opportunities and risks of diversity and the specific challenges for people with a migration background and younger and older employees. In this context, both one’s own experiences and sensitivity, as well as the position of the company and previous measures, seem to be relevant and interact with one another, predicting attitudes toward diversity. Different patterns emerge among respondents when they interact a lot or little with people with a migration background and when they identify more or less with their company. These findings are broadly consistent with existing findings on attitudes toward cultural diversity (van Dick and Stegmann, 2016; Gutentag et al., 2018; Genkova and Schreiber, 2019), implying that STEM leaders’ and employees’ diversity attitudes are quite comparable to those in other industries, although stereotypes of intelligent but unemphatic men in STEM professions suggest the opposite. However, inter-individual differences between the respondents seem to be due to the work context and systematic deficits in knowledge and competence in dealing with cultural diversity and less to the affiliation to the STEM industry, or particularly strong matches with the stereotypical STEM. Managers who rarely work with people with a migration background show fewer specific skills in dealing with diversity, are less sensitive to diversity challenges, and represent a more culture-blind perspective. They justify why diversity is important by arguing that all people are equal, but because they treat everyone equally, they do not have a problem with diversity.

This result is in line with the results of Genkova and Schreiber (2019), who found many managers from all industries to not even notice the challenges in their employees’ everyday work. Those who showed these more negative attitudes in this study also agreed that applicants must speak German to belong to the organization. Pehrson et al. (2009) used a cross-sectional study to show that the connection between national identity and prejudices against foreigners is stronger when nationality is defined by ethnicity or language compared to when nationality is defined by citizenship. The fact that the participants speak from a company perspective and link reservations to operational issues suggests that identification with the company can also lead to a more negative and undifferentiated attitude toward people with a migration background if the German language is perceived as a relevant part of the corporate culture. Future studies should further elaborate on this result.

Those who have contact with people with a migration background are not necessarily more sensitive to people with a migration background or more competent in dealing with them. This awareness is only present if diversity measures have already been implemented. A clear positioning of companies as “pro-diversity” with a high level of identification with the company means that positive and differentiated attitudes are more likely to be adopted, which corresponds to the results of Ashikali and Groeneveld (2015). If there is a high level of identification and a lacking or negative positioning of the company, the term “them” is used more regularly talking about people with a migration background. Apparently, people with a migration background are perceived as external to the organization or team. Based on Mummendey and Wenzel (1999), this assessment can be traced back to the ingroup projection hypothesis, which states that people experience a superordinate group, such as a company, as more homogeneous and similar to themselves than it actually is. Gutentag et al. (2018) show that on the one hand, prejudice and xenophobia can be the reason for culture blindness, but also the feeling of being overwhelmed by the complexity of the topic. In fact, most of the participants seem to have realized that the topic of cultural diversity is present but very complex and that their existing knowledge is not sufficient. Employees in particular feel inadequately supported by the organization and interact in a complex, diverse social environment unaware of risks or potentials. A previous study by Genkova and Schreiber (2019) points out that such negative experiences without support or explanation are very likely to lead to problematizing diversity. This could explain the tendency among employees to ignore cultural diversity in particular and just try to avoid conflicts.

It is also pointed out that dealing with diversity is not taught in STEM courses, although it is not mentioned that other courses do this as standard. Respondents seem to differ in how they deal with this uncertainty. This fits with the tendency of some of the STEM leaders surveyed to legitimize their own views with the attitude of management or the company, which can be taken as a sign of insecurity. Other managers (particularly from larger companies) solve the problem by continuing their education. This may be due to better access to further training opportunities in large companies, but also because there appears to be a greater variety of specialist areas at management level and often a clear company line. Thereby, managers are more likely to devote time and attention to the topic of cultural diversity, if it is subjectively anchored in the corporate identity. Those managers who are more aware of the problems and the challenges are also convinced that leadership in general and diversity leadership in particular requires special training or further training. This supports the connection between company positioning, further training opportunities and the experience of a lack of competence.

It is emphasized that junior managers and employees often do not meet the requirements of a diverse working environment and go through a not unproblematic try-and-error process until they can work without problems. This also corresponds to the cross-industry observations of Barmeyer et al. (2019). A central result of this study is therefore that both companies and universities need to take greater account of cultural and age diversity in order to be able to meet the requirements of a diversifying society.

The analysis of the interviews also shows that agile working and diversity sensitivity do not necessarily go hand in hand. McCallaghan et al. (2020) conducted a cross-sectional study to demonstrate that servant leadership, a core concept of agile frameworks, is positively associated with employees’ diversity attitudes. However, they also point out that they operationalize diversity attitudes only as an instrumental component (i.e., whether respondents believe diversity represents an economic benefit) and do not capture diversity sensitivity. Like Ashikali and Groeneveld (2015), they show that an employee-oriented leadership style enables the manager to pass on their attitudes, which can have advantages and disadvantages. On the one hand, equality is firmly anchored in both agile frameworks and transformational leadership style (Pusenius, 2019). On the other hand, neither concept addresses other facets of diversity management. The IT people, who describe their leadership style as agile emphasize equality but have no in-depth knowledge or awareness of the opportunities and risks of cultural diversity. Although the rest of the mechanisms outlined in this work could also work for this attitude, a connection seems obvious and further studies should address a connection between agile methods and diversity attitudes, especially given the increasing prevalence of agile working methods.

### Limitations

Women and men showed no differences in response behavior, but a separate consideration of possible connections between attitudes and experiences was not undertaken. As all participating employees were working in some form of team structure rather than alone, results might not be transferable to STEM-scientists working mostly on their own. Although the perspectives of managers with a migration background were considered separately, it is likely that this study cannot be transferred to other groups with regard to this aspect either. Future studies should resolve this problem and specifically address women and people of different genders as well as people with a migration background, also in order to take possible effects of intersectionality into account. Since no suitable comparative material is available, no statements can be made about the extent to which the participants provide a typical or generalizable picture of the STEM industry. Neither is it possible to draw any conclusions about actual skills and characteristics, as this would have required an additional survey of employees and customers, for example. However, the results suggest that the participants’ deficits in terms of diversity awareness and competencies are mainly caused by the work context and lack of training, rather than by a specific predisposition of people in STEM professions. In addition, for further studies, it seems promising not only to ask about perspectives and opinions, but to have them substantiated by means of situational questions. While some respondents backed up their discussions with many examples, others were very reluctant, especially when it came to negative experiences.

### Conclusion

Despite its limitations, the present study was able to explore relevant connections for further research in the field of diversity and diversity management. It should be emphasized in particular that managers are by no means mere intermediaries between an overarching diversity strategy and employees, as studies such as the work of Ashikali and Groeneveld (2015) implicitly assume. The perception of the dimensions of age and cultural diversity appears to be a crucial precondition for fighting structural discrimination, such as lower payments for older migrants over the life course. Future studies are encouraged to consider special aspects of the perspectives toward diversity, i.e., identification with the company and companies’ diversity cultures for managers, as well as feeling of support and preparation of employees. In practice, the results of this study implicate that diversity management in STEM is very relevant for the support of managers and employees and that workforce must be equipped with the necessary knowledge and understanding to be able to act efficiently and appropriately. Furthermore, the present results indicate that agile working does not create diversity awareness. Even university graduates cannot be assumed to have experience in this area.

## Data availability statement

The raw data supporting the conclusions of this article will be made available by the authors, without undue reservation.

## Ethics statement

Ethical review and approval was not required for the study on human participants in accordance with the local legislation and institutional requirements. The patients/participants provided their written informed consent to participate in this study.

## Author contributions

PG was responsible for study design, project planning and coordination, evaluating results, and supervising the analyses and writing process. HS was involved in conducting interviews and analyses and writing the first draft of the manuscript. All authors contributed to the article and approved the submitted version.

## Conflict of interest

The authors declare that the research was conducted in the absence of any commercial or financial relationships that could be construed as a potential conflict of interest.

## Publisher’s note

All claims expressed in this article are solely those of the authors and do not necessarily represent those of their affiliated organizations, or those of the publisher, the editors and the reviewers. Any product that may be evaluated in this article, or claim that may be made by its manufacturer, is not guaranteed or endorsed by the publisher.

## References

[ref1] Anger C. Koppel O. Plünnecke A. (2018). MINT-Früjahrsreport 2018: MINT – Offenheit, Chancen, Innovationen. Available at: https://www.arbeitgeber.de/www/arbeitgeber.nsf/res/MINT-Fruehjahrsreport_2018.pdf/$file/MINT-Fruehjahrsreport_2018.pdf (Accessed August 8. 2022).

[ref2] Ashikali T. Groeneveld S. (2015). Diversity management in public organizations and its effect on employees’ affective commitment. Rev. Public Pers. Adm. 35, 146–168. doi: 10.1177/0734371X13511088

[ref3] Auferkorte-Michaelis N. Linde F. (eds.) (2018). Diversität lernen und lehren: Ein Hochschulbuch. Germany: Verlag Barbara Budrich.

[ref4] Bader A. K. Kemper L. E. Froese F. J. (2019). Who promotes a value-in-diversity perspective? A fuzzy set analysis of executives’ individual and organizational characteristics. Hum. Resour. Manag. 58, 203–217. doi: 10.1002/hrm.21946

[ref5] Barmeyer C. I. Bausch M. Moncayo D. (2019). Cross-cultural management research: topics, paradigms, and methods—a journal-based longitudinal analysis between 2001 and 2018. Int. J. Cross-cult. Manag. 19, 218–244. doi: 10.1177/1470595819859603

[ref6] Bengtsson M. (2016). How to plan and perform a qualitative study using content analysis. NursingPlus Open 2, 8–14. doi: 10.1016/j.npls.2016.01.001

[ref001] Bissles S. Sackmann S. Bissels T. (2001). Kulturelle Vielfalt in Organisationen. Ein blinder Fleck muss sehen lernen. Soziale Welt (54), Artikel 4, 403–426.

[ref7] Biswas K. Boyle B. Bhardwaj S. (2021). “Impacts of supportive HR practices and organisational climate on the attitudes of HR managers towards gender diversity – a mediated model approach.” in Evidence-Based HRM: A Global Forum for Empirical Scholarship. *Vol.* 9, 18–33.

[ref8] Buttner E. H. Lowe K. B. Billings-Harris L. (2006). The influence of organizational diversity orientation and leader attitude on diversity activities. J. Manag. Issues 18, 356–371.

[ref004] Canagarajah S. (2018). Materializing ‘Competence’: Perspectives From International STEM Scholars. Mod. Lang. J. 102, 268–291. doi: 10.1111/modl.12464

[ref9] Cox T. (1991). The multicultural organization. Acad. Manag. Perspect. 5, 34–47. doi: 10.5465/AME.1991.4274675

[ref10] Charta der Vielfalt (2019). Zukunftsfaktor Vielfalt: Diversity Management für den Mittelstand. Available at: https://www.charta-der-vielfalt.de/fileadmin/user_upload/Studien_Publikationen_Charta/Charta_der_Vielfalt_-_KMU-Brosch%C3%BCre_2020.pdf (Accessed August 8. 2022).

[ref11] Eger L. Indruchová Z. (2014). Diversity management - perceptions and attitudes by. Czech Managers. 17:17. doi: 10.15240/tul/001/2014-1-006

[ref12] Ferris G. R. Frink D. D. Galang M. C. (1993). Diversity in the workplace: the human resources management challenges. *Human*. Resour. Plan. 25, 41–51.

[ref13] Genkova P. (2019). *Interkulturelle Wirtschaftspsychologie* [1. Auflage]; October 31, 2019–November 2, 2019. Germany: Springer-Lehrbuch.

[ref14] Genkova P. Ringeisen T. (2016). Handbuch Diversity Kompetenz. Wiesbaden: Springer Fachmedien Wiesbaden.

[ref15] Genkova P. Schreiber H. (2019). “Diversity potentials in organizations.” in *IACCM Research Conference: Intercultural Competencies for a disruptive VUCA World: Exploring creativity, innovation, resilience & resistance in intercultural research, training & management*. eds. B. C. Venegas, C. Debray and J. Vakkayil (Paris: IACCM).

[ref16] Goldman B. M. Slaughter J. E. Schmit M. J. Wiley J. W. Brooks S. M. (2008). Perceptions of discrimination: a multiple needs model perspective. J. Manag. 34, 952–977. doi: 10.1177/0149206308318613

[ref17] Gutentag T. Horenczyk G. Tatar M. (2018). Teachers’ approaches Toward cultural diversity predict diversity-related burnout and self-efficacy. J. Teach. Educ. 69, 408–419. doi: 10.1177/0022487117714244

[ref002] Hilmer J. (1969). Grundzüge einer pädagogischen Theorie der Bewegungsspiele. Ein Beispiel zur Didaktik der Leibeserziehung. Hannover: Schroedel.

[ref003] Kemper T. (2010). Migrationshintergrund – eine Frage der Definition! Die Deutsche Schule 102, 315–326.

[ref18] Koller K. Rudolph C. (2017). *Gender Mainstreaming und Diversity in der (akademischen) MINT-Weiterbildung: Konzepte, Prozesse und Befunde*. OTHmind–BMBF-Verbundprojekt.

[ref19] Krell G. (2013). “Vielfältige Perspektiven auf Diversity: Erkunden, enthüllen, erzeugen,” in Diversity entdecken. Reichweiten und Grenzen von Diversity Policies an Hochschulen. eds. Schmidbaur M. Wolde A. Bender S.-F. (Weinheim: Beltz Juventa), 61–78.

[ref20] Kundu S. C. Mor A. (2017). Workforce diversity and organizational performance: a study of IT industry in India. Empl. Relat. 39, 160–183. doi: 10.1108/ER-06-2015-0114

[ref21] Kunze F. Boehm S. Bruch H. (2013). Organizational performance consequences of age diversity: inspecting the role of diversity-friendly HR policies and top managers’ negative age stereotypes. J. Manag. Stud. 50, 413–442. doi: 10.1111/joms.12016

[ref22] Madera J. M. Dawson M. Neal J. A. (2017). Managers’ psychological diversity climate and fairness: The utility and importance of diversity management in the hospitality industry. J. Hum. Resour. Hosp. Tour. 16, 288–307. doi: 10.1080/15332845.2017.1253442

[ref23] Mayring P. (2019). Qualitative Inhaltsanalyse–Abgrenzungen, Spielarten, Weiterentwicklungen. Forum Qual. Sozialforsch./ Forum Qual. Soc. Res. 20, 1–14. doi: 10.17169/fqs-20.3.3343

[ref24] McCallaghan S. Jackson L. T. B. Heyns M. M. (2020). Servant leadership, diversity climate, and organisational citizenship behaviour at a selection of south African companies. J. Psychol. Afr. 30, 379–383. doi: 10.1080/14330237.2020.1821310

[ref25] Mummendey A. Wenzel M. (1999). Social discrimination and tolerance in intergroup relations: reactions to intergroup difference. Pers. Soc. Psychol. Rev. 3, 158–174. doi: 10.1207/s15327957pspr0302_4 15647144

[ref005] Pehrson S. Brown R. Zagefka H. (2009). When does national identification lead to the rejection of immigrants? Cross-sectional and longitudinal evidence for the role of essentialist in-group definitions. Br. J. Soc. Psychol. 48, 61–76. doi: 10.1348/014466608X288827 18302807

[ref006] Pusenius K. (2019). Agile Mindset in the Workplace. Mving towards Organizational Agility. Master´s Thesis. Turku University of Applied Sciences.

[ref26] Rosken A. (2016). “Konzept Diversity Management–Definition, Abgrenzung und Beurteilung,” in Handbuch Diversity Kompetenz. eds. Genkova P. Ringeisen T. (Wiesbaden: Springer Fachmedien Wiesbaden), 61–73.

[ref27] Sanchez D. (2018). Racial and structural discrimination toward the children of indigenous Mexican immigrants. Race Soc. Probl. 10, 306–319. doi: 10.1007/s12552-018-9252-2

[ref28] Schein E. H. (2010). Organizaitonal Culture and Leadership (4th edn.). Mahwah: Erlbaum.

[ref29] Schneid M. Isidor R. Steinmetz H. Kabst R. (2016). Age diversity and team outcomes: a quantitative review. J. Manag. Psychol. 31, 2–17. doi: 10.1108/JMP-07-2012-0228

[ref007] Schwaber K. Sutherland J. (2020). The Scrum Guide. The Definitive Guide to Scrum: The Rules of the Game. 2. Edt., Online available from https://www.scrumguides.org/docs/scrumguide/v2020/2020-Scrum-Guide-US.pdf#zoom=100 (Accessed August 8. 2022).

[ref30] Stegmann S. (2011). Engaging with Diversity of social Units. Frankfurt: Deutsche Nationalbibliothek.

[ref31] Stypińska J. Gordo L. R. (2018). Gender, age and migration: an intersectional approach to inequalities in the labour market. Eur. J. Ageing 15, 23–33. doi: 10.1007/s10433-017-0419-2, PMID: 29531512 PMC5840085

[ref32] Tajfel H. Turner J. C. (1979). “An integrative theory of intergroup conflict,” in The social Psychology of Intergroup Relations. eds. Austin W. G. Worchel S. (Utah: Brooks/Cole), 33–47.

[ref33] Thomas A. Simon P. (2007). “Interkulturelle Kompetenz,” in Enzyklopädie der Psychologie: Vol. 3. Anwendungsfelder der kulturvergleichenden Psychologie: Themenbereich C Theorie und Forschung. Serie 7 Kulturvergleichende Psychologie. eds. Trommsdorff G. Konradt H.-J. (Göttingen: Hogrefe), 135–177.

[ref34] Torres L. Driscoll M. W. Voell M. (2012). Discrimination, acculturation, acculturative stress, and Latino psychological distress: a moderated mediational model. Cultur. Divers. Ethnic Minor. Psychol. 18, 17–25. doi: 10.1037/a0026710, PMID: 22250895 PMC3340887

[ref36] van Dick R. Stegmann S. (2016). “Diversity, Social Identity und Diversitätsüberzeugungen,” in Springer Reference Psychologie. Handbuch Diversity Kompetenz. eds. Genkova P. Ringeisen T. (Berlin: Springer), 3–16.

[ref37] van Knippenberg D. Haslam S. A. Platow M. J. (2004). Unity through diversity: value-in-diversity beliefs, work group diversity, and group identification. Group Dyn. Theory Res. Pract. 11, 207–222. doi: 10.1037/1089-2699.11.3.207

[ref38] van Knippenberg D. Haslam S. A. (2003). “Realizing the diversity dividend: exploring thesubtle interplay between identity, ideology, and reality,” in Social Identity at Work: Developing Theory for Organiza-tional Practice. eds. Haslam S. A. van Knippenberg D. Platow M. J. Ellemers N. (London: Psychology Press), 61–77.

[ref39] van Knippenberg D. Schie E. C. M. (2000). Foci and correlates of organizational identification. J. Occup. Organ. Psychol. 73, 137–147. doi: 10.1348/096317900166949

[ref40] Walker S. S. Corrington A. Hebl M. King E. B. (2021). Subtle discrimination overtakes cognitive resources and undermines performance. J. Bus. Psychol. 37, 311–324. doi: 10.1007/s10869-021-09747-2

[ref41] Wang J. Cheng G. H.-L. Chen T. Leung K. (2019). Team creativity/innovation in culturally diverse teams: a meta-analysis. J. Organ. Behav. 40, 693–708. doi: 10.1002/job.2362

[ref42] Wildermuth C. Gray S. (2005). Diversity Training. Alexandria, VA: ASTD Press.

[ref43] Yadav S. Lenka U. (2020). Diversity management: a systematic review. Equal., Divers. Incl. Int. J. 39, 901–929. doi: 10.1108/EDI-07-2019-0197

